# Availability of opioid agonist treatment and critical incidents in Forensic Clinics for Dependency Diseases in Germany

**DOI:** 10.3389/fpsyt.2022.961549

**Published:** 2022-09-07

**Authors:** Sven Reiners, Annette Opitz-Welke, Norbert Konrad, Alexander Voulgaris

**Affiliations:** ^1^Krankenhaus des Maßregelvollzuges Berlin, Forensic Psychiatric Hospital Berlin, Berlin, Germany; ^2^Institute of Forensic Psychiatry, Charité—Universitätsmedizin Berlin, corporate member of Freie Universität Berlin and Humboldt-Universität zu Berlin, Berlin, Germany; ^3^Department of Psychiatry and Psychotherapy, Prison Hospital Berlin, Berlin, Germany; ^4^Institute of Sex Research, Sexual Medicine and Forensic Psychiatry, Universitätsklinikum Hamburg-Eppendorf, Hamburg, Germany

**Keywords:** opioid agonist treatment, critical incidents, escape, violent behavior, forensic psychiatry

## Abstract

**Background:**

Prevalence of substance use disorders, especially opioid use disorders, is high in patients admitted into forensic psychiatric settings. Opioid agonist treatment is a safe, well-established, and effective treatment option for patients that suffer from opioid dependence. Surprisingly, data on the availability and practice of opioid agonist treatment (OAT) options in German Forensic Clinics for Dependency Diseases is rare. Furthermore, essential data on the prevalence of critical incidents such as violent behavior, relapse, or escape from the clinic are missing for this particular treatment setting.

**Materials and methods:**

We conducted an observational study on all forensic addiction treatment units in Germany (Sect. 64 of the German Criminal Code). A questionnaire on the availability and practice of OAT was sent to all Forensic Clinics for Dependency Diseases in Germany. Following items were assessed: availability and the total number of patients that received an OAT in 2018, available medication options, specific reasons for start and end of OAT, number of treatments terminated without success, number of successful treatments, and critical incidents such as violent behavior, relapse, escape and reoffending. We compared the forensic clinics that offered OAT with those that did not offer this treatment option. The data were analyzed descriptively. Mean and standard deviation was calculated for metric scaled variables. For categorical variables, absolute and relative frequencies were calculated. The two groups (OAT vs. Non-OAT institutions) were compared concerning the given variables by either using Fishers exact test (categorical variables), *t*-test (normally distributed metric variables), or Wilcoxon-test (metric variables not normally distributed).

**Results:**

In total, 15 of 46 Forensic Clinics for Dependency Diseases participated in the study (33%). In total, 2,483 patients were treated in the participating clinics, 18% were relocated into prison due to treatment termination, and 15% were discharged successfully in 2018. 275 critical incidents were reported: violence against a patient (4%), violence against staff (1.6%), escape (4.7%) and reoffending in (0.5%). In seven clinics treating 1,153 patients, an OAT was available. OAT options in forensic clinics were buprenorphine/naloxone, buprenorphine, methadone, and levomethadone. Regarding critical incidents and successful discharge, no differences were detected in the clinics with or without an OAT. In the clinics that offered an OAT, we found a significantly higher rate of treatment termination without success (*p* < 0.007) in comparison to clinics without an OAT program. Ninety-nine patients received an OAT, and this treatment was ended due to illegal drug abuse (57%), refusal to give a urine drug sample (71%), and cases where the OAT was given away to other patients (85%).

**Conclusion:**

In Forensic Clinics for Dependency Diseases in Germany, OAT is not available in every institution, and thus, access is limited. Critical incidents such as violent behavior against staff or patients and escape are not uncommon in these forensic treatment settings. Further studies are needed to enhance the understanding of OAT practice and the risks for patients and staff.

## Introduction

Addiction therapy in forensic clinics for dependency disorders (FCDD) is an ongoing controversial topic of discussion regarding the necessity, quality of care, effectiveness, and mode of implementation in Germany (1–4). It is known that substance abuse disorders are highly prevalent in forensic psychiatric and prison contexts and play an essential role in crime, the risk for reoffending, violent behavior, and mental disorders (5–7).

While comprehensive data describing differences in forensic psychiatric care, admission numbers over time, and legal frameworks in European countries (8–10) exist, data on specific FCDD or available treatment options for (comorbid) substance abuse disorders in forensic psychiatry are lacking on a national and on an international level (11, 12). After 30 years of deinstitutionalization with a reduction of general psychiatric bed capacity, a trend toward reinstitutionalization with higher admission rates into forensic psychiatric care is evident (13). In this aspect, higher rates of comorbid substance misuse are discussed as one potential reason for this development (8).

In Germany, specialized psychiatric-psychotherapeutic care is offered in FCDD to offenders that committed a crime in combination with a substance use disorder. These FCDD are typically separated from general forensic psychiatric care, and bed capacity in FCDD in Germany is continuously rising, with 1,230 patients in the year 1994 and 4,500 patients in treatment in the year 2021 (2, 14–16). The rationale for these specific treatment institutions is that some committed crimes are supposedly connected to an individual substance use disorder and by offering an intensive treatment possibility for these patients, in theory, the risk for relapse and re-incarceration after release can be diminished. Studies suggest that successful treatment participation is associated with higher rates of abstinence and fewer criminal relapses (3). But, it is important to note that studies repeatedly described a rate of about 50% when it comes to unsuccessful treatment termination in FCDD (2, 17). Unsuccessful treatment termination leads to a transfer into general prison facilities, where OAT is (often) available, but specific psychotherapeutic interventions or group therapy is lacking. These developments underline the importance of intensifying research activities to understand better what happens in these specific forensic treatment settings.

The World Health Organization (WHO) and the German Society for Addiction Medicine are clear when recommending opioid agonist treatment (OAT) as a first-line, practical, and evidence-based treatment option for opioid addiction with a positive influence on mortality, drug use, and treatment compliance (18–24). Still, to the best of our knowledge, it is unknown to what extent opioid agonist treatment is available to the patients treated in FCDD nationwide.

As stated above, successful treatment of substance use disorders may positively influence the risk for dangerous and impulsive patient behavior. In FCDD, all patients admitted are diagnosed with at least one moderate to severe substance use disorder. Thus, information on critical incidents such as violent behavior or escape/absconding during treatment is a relevant to better understand what patients and personnel experience during the process and what they must cope with in this specific forensic treatment setting.

## Aims of our study

We aimed to describe the availability and clinical practice of OAT in FCDD nationwide. Due to our clinical experience in the field, we hypothesized that OAT availability and implementation would be largely heterogeneous in Germany. The leading author SR was the chief doctor of the FCDD in Berlin, Germany, so general information on OAT options in FCDD was available to some extent. The lack of scientific data on this relevant topic (12) in forensic psychiatric care is well-known in Germany and with this in mind, this study was conducted. In addition, we were interested in the frequency and typology of specific critical incidents during treatment episodes and the discharge mode in the FCDD in the year 2018.

## Materials and methods

### Study setting

In German law, under specific circumstances, courts can apply a dependency treatment order to offenders who suffer from a substance use disorder and commit an unlawful act (Section 64 German criminal code). Preconditions for this treatment order are offenses above a certain threshold and a direct or indirect connection to the offender's substance use disorder (e.g., intoxication, offense to finance the substance abuse). During the trial, the judge orders an expert witness with particular expertise in forensic psychiatry to report on the diagnosis and the legal and treatment prognosis regarding specialized therapy in an FCDD. Only patients with a favorable treatment prognosis should enter therapy in the FCDD, with an average length of stay of 2 years.

### Study design

We conducted an observational study including all FCDD in Germany for 2018. *Via* postal survey, all chief doctors of the existing 46 FCDD in Germany were contacted and invited to participate in our study and received the questionnaire. It is important to note that the questionnaire was anonymous in nature. This means that survey responders and their institution were kept anonymous and thus, no data regarding the specific location of the FCDD was attained. This was decided in order to ensure a high participation rate. After 3 months, follow-up letters were sent *via* email to increase the response rate. How the chief doctors generated the specific information in their FCDD was not asked for. It is common in Germany, that FCDD have their own administrative database systems with which the questionnaire can be completed.

### Questionnaire information

The questionnaire was two pages long and asked for 13 items. Items included detailed information regarding the clinical practice with OAT in the local FCDD, such as availability, year of availability and total number of patients that received an OAT in 2018, available medication options for OAT, specific reasons for starting and ending an OAT, total number of treatment terminations without success, number of successful treatments and the total number of critical incidents such as violent behavior against staff, violent behavior against other patients, drug or alcohol relapse, escape from the clinic, escape during relaxation of security measures, and occurrence of a new offense during ongoing treatment. Information on diagnosis was classified using the International Classification of Diseases ICD-10.

### Statistical analyzes

The data were analyzed descriptively. Mean and standard deviation was calculated for metric scaled variables. For categorical variables, absolute and relative frequencies were calculated. The two groups (FCDD with OAT vs. FCDD without OAT) were compared concerning the given variables by either using Fishers exact test (categorical variables), *t*-test (normally distributed metric variables), or Wilcoxon-test (metric variables not normally distributed). For all analyzes, *p* < 0.05 was considered significant. We performed all analyzes using IBM SPSS Statistics, version 25.0.

## Results

In total, 15 of the available 46 FCDD in Germany participated in our survey (33%). Due to the anonymity of the study, there was no information regarding which FCDD participated or in how far they differed from the FCDD that did not respond. The participating FCDD treated 2,483 patients in 2018. Of these, 444 (18%) patients were relocated into prison due to treatment termination, and 379 (15%) were discharged after completing the treatment program. Critical incidents were reported in 275 cases (see Table 1).

**Table 1:** Reported critical incidents in Forensic Clinics for Dependency Diseases in Germany in 2018.

**Critical incidents**	**Total**
Violent behavior against another patient	103 (37.45%)
Violent behavior against staff	39 (14.18%)
Offense during relaxation of security measures	12 (4.36%)
Escape during relaxation of security measures	118 (42.90%)
Escape from the clinic	3 (1.09%)
Total	275 (100%)

In seven of the 15 participating FCDD (47%), an OAT program was available. In these seven FCDD, 1,153 patients were treated during 2018. Regarding specific characteristics and medical OAT options, see Table 2.

**Table 2:** Patient numbers and available OAT options in seven Forensic Clinics for Dependency Diseases in Germany in 2018.

	**FCDD 1**	**FCDD 2**	**FCDD 3**	**FCDD 4**	**FCDD 5**	**FCDD 6**	**FCDD 7**	**Total**
Patients total	369	61	152	122	244	111	94	1,153
OAT program (year)	2011	–	2007	2015	2017	2018	2001	
**Patients with OAT**	34 (9.21%)	13 (21.31%)	4 (2.63%)	13 (10.65%)	13 (5.32%)	20 (18.01%)	2 (2.12%)	99 (8.58%)
- Methadone	+	+	+	+	+	–	–	
- Levomethadone	+	+	+	+	+	+	–	
- Buprenorphine	–	+	+	+	+	+	+	
- Buprenorphine/Naloxone	+	+	–	+	+	–	–	
- Morphine	–	+	–	–	–	–	–	
- Diamorphine	–	–	–	–	–	–	–	

In all seven FCDDs offering an OAT program, patients were included with an existing diagnosis of opioid substance addiction (ICD-10 F11.2) and also in combination with other comorbid substance addiction disorders (ICD-10: F1X.2) or due to the diagnoses of a polyvalent substance use disorder (ICD-10: F19.2). All seven FCDD with an OAT program started or continued an OAT when the patient had already received an OAT before admission to the FCDD. Four of the seven FCDDs offered to start a new OAT after the initial diagnostic phase of the treatment process. Moreover, two of the seven FCDDs offered to start an OAT at the end of the treatment process when security measures were loosened.

The OAT program ended due to the following reasons. In four FCDDs, the OAT was ended due to illegal drug or opioid abuse (57%), in five FCDDs due to the refusal to give a urine drug sample (71%), and in six FCDDs due to cases where the OAT was given away to other patients (85%). Low patient compliance during the treatment process was a reason for one of the FCDDs. For more detailed information concerning the reasons for OAT termination, see Table 3.

**Table 3:** Clinical practice for ending OAT in the seven Forensic Clinics for Dependency Diseases offering OAT.

	**FCDD 1**	**FCDD 2**	**FCDD 3**	**FCDD 4**	**FCDD 5**	**FCDD 6**	**FCDD 7**
Illegal opioid abuse	X		X	X	X		
Illegal drug abuse	X		X		X	X	
No urine sample	X		X	X	X	X	
Giving away OAT	X	X	X	X	X	X	
No compliance					X		
Patients decision							X

We formed subgroups and compared the FCDD with and without an established OAT program regarding critical incidents and discharge mode. In successful treatment, no differences were detected in the clinics with or without an OAT program. In the clinics that offered an OAT, we detected a significantly higher rate of treatment termination without success (*p* < 0.007) in comparison to clinics without an OAT program (see Table 4).

**Table 4:** Comparison of FCDD with and without an OAT program in 2018 (mean with standard deviation).

	**FCDD without OAT**	**FCDD with OAT**	***p* overall**
Patients per FCDD	166 (±87)	165 (±107)	0.976
Treatment terminations	20.6 (±16.7)	42.9 (±34.5)	0.160
Successful treatments	28.1 (±13.4)	22.0 (±8.8)	0.310
Violence against patients	9.3 (±8.67)	7.6 (±7.3)	0.723
Violence against staff	3.38 (±4.6)	2.4 (±2.1)	0.613
Escape from clinic	0.38 (±0.74)	0.00 (±0.00)	0.197
Escape during relaxation of security measures	8.75 (±6.43)	8.00 (±12.1)	0.894
Offense during relaxation of security measures	0.38 (±0.74)	1.80 (±2.49)	0.275
Treatment terminations in relation to total treatments	11.0 (±7.37)	25.6 (±9.20)	0.007
Treatment success in relation to total treatments	17.4 (±14.0)	15.8 (±6.75)	0.780

In the seven FCDD offering OAT in 2018, 99 patients were included in the OAT program (8.5%). Of these, 25 were relocated into prison due to treatment termination, and in nine cases, successful treatment progress was reported. Including all participating FCDD of our study, merely 3.9% of all 2,483 patients received an OAT.

## Discussion

Although merely 33% of FCDD responded to our postal survey, 2,483 patients were included in the study, and thus more than 50% of all patients treated in FCDD in Germany in 2018 were represented in our sample (2). Of 823 patients that ended the therapy in 2018, 53% were relocated to prison, which aligns with the published data on unsuccessful treatments (2, 17). As expected, variability in the clinical practice regarding OAT is high and availability relatively low in FCDD in Germany. Only seven out of 15 FCDD offered an OAT program, and merely 8.6% (2.6–21.3%) of patients in these FCDD received an OAT. Berthold and Riedemann demonstrated in a cross-sectional study including 2,046 patients that in the year 2019, 32% of the patients in FCDD had a primary diagnosis of a polyvalent substance dependency disorder (ICD-10: F19.2) and 10% had a primary opioid dependency disorder (ICD-10: F11.2). Also, in 2019, 19% experienced an OAT before entering the FCDD (25). This possible under treatment of patients in need of OAT supports the often described “clinic-effect” — the influence of the individual setting and treatment possibilities in each FCDD—and its consequences for treatment outcome (25–27).

In comparison, in Western European and German prison facilities availability of OAT is higher (7, 28), which is surprising given that FCDD are specialized treatment centers for substance use disorders. A possible explanation may be that a common goal in FCDD is often total abstinence of all substances and that an OAT may not be considered a sufficient therapy success (1, 25, 29, 30). In our opinion, the often described positive effects of OAT on general health and recidivism are in stark contrast to the specific practice in FCDD (18–25, 31–39), and standardization of available treatment possibilities is recommended.

Regarding the clinical practice of OAT, only four FCDDs offered a new implementation of OAT after an initial diagnostic phase. In only two FCDDs, an OAT was started at the end of the therapeutic process. In our opinion, this reflects a rather limited access to OAT in general, and also in the FCDD that offer this well-established and, in general practice, common treatment option (18–24). Further, these results are relevant insofar that the literature suggests two critical points in the treatment process where premature terminations are common: at the beginning and at the end of the therapeutic process, where patients are confronted with a stepwise re-entry into society and a loosening of security measures (29). Reasons for ending an OAT during the process also varied between the different FCDDs. Discontinuing OAT due to refusal of providing a urine sample or engagement in illegal drug use are not evidence-based reasons for terminating the OAT, and rather enhances individual risk for relapse, criminal recidivism and overdose symptoms. Note that specific information on the individual therapeutic process and context regarding the discontinuation of the OAT was not recorded in the study. As expected, methadone, levomethadone, and buprenorphine were frequently prescribed as OAT, while morphine was only available in one FCDD, and diamorphine was not prescribed in any of the participating FCDD. To the best of our knowledge, comparable data from other countries are missing.

When comparing the FCDD with and without an OAT program, we detected no differences in the number of critical incidents. In total, escape during stepwise relaxation of security measures was identified in 4.7%, which is lower when compared to available data from 2012 covering the years 2001 through 2009 and analyzing 994 cases in Regensburg, Bavaria, where 15% escaped at least once during the treatment process (40). In his study, Hartl found that 2.5% of the patients demonstrated violent behavior against staff and 6% against other patients and that 6% reoffended during the therapy, which is also higher in comparison to our results. On the one hand, this supports the above-mentioned “clinic effect” and the observation of high heterogeneity between the different FCDD (40). On the other hand, this may result from improved security measures. Although the current numbers are lower than the limited data for the past suggests, critical incidents are still part of clinical reality in forensic psychiatric institutions, and we believe that implementing more differentiated treatment programs such as OAT could lead to a more individualized and thus optimized therapy.

Interestingly, regarding the treatment process, FCDD offering OAT had a significantly higher rate of premature treatment terminations, which was not expected due to the often discussed positive effects of OAT (25, 39). It may be possible that in federal states where FCDD offers OAT, the admission practice is more open regarding patient groups that suffer from especially severe substance use disorders, which may lead to a more complicated treatment process. It is important to note that our data did not ask for the severity of substance use or comorbid mental disorders and did not include information on the specific reasons for treatment termination. It is relevant to note that we present aggregate rates of all patients in FCDD with different substance use disorders, not only opioid use disorders, so the true association between the availability of an OAT program and its possible (positive) effects on critical incidents or treatment outcome cannot be explained by our data. Future studies could be conducted as cohort studies with a more precise focus on opioid use disorders, their specific rate for critical incidents and with controlling for potentially confounding factors (e.g., comorbid mental or substance use disorders).

In 2009, Schalast formulated that OAT could and should be an appropriate treatment option for patients in FCDD and that certain flexibility is needed for its implementation (41). Thirteen years later, our data suggest that OAT programs are unavailable nationwide in FCDD. Thus, patients treated in FCDD are at a disadvantage compared to patients in general society and even those in prison. More research and consistent data are necessary to better understand the differences in the clinical practice and to optimize treatment options for patients receiving a court order for therapy in FCDD.

### Limitations

Several limitations have to be considered when interpreting our findings. No individual sociodemographic data or data on offense type, comorbid mental disorders such as personality disorders, psychosis or affective disorders, the severity of the symptoms, and other medication was available. Also, data on the reasons for the critical incidents and treatment termination was not assessed. The retrospective design may have led to various biases, and the obtained data, in general, did not allow for in-depth statistical analyzes. A larger and more specific sample would be necessary to better understand critical incidents in FCDD because these incidents are still rare. Our results are temporal and cannot explain current treatment options in FCDD.

In our opinion, the presented data is vital in the ongoing discussion about reforming the clinical and legal practice in Germany regarding the criminal code 64 and general addiction treatment in forensic psychiatric settings.

## Data availability statement

The raw data supporting the conclusions of this article will be made available by the authors, without undue reservation.

## Ethics statement

According to current legal regulations, the study was approved by the local Ethic Committee at Charité–Universitätsmedizin Berlin.

## Author contributions

SR, AO-W, AV, and NK designed the study and had full access to all the data in the study and take responsibility for the integrity of the data and the accuracy of data analysis. SR collected the data. AV, NK, and AO-W analyzed and interpreted the data. SR and AV wrote the final draft of the manuscript. All authors contributed to the article and approved the submitted version.

## Conflict of interest

The authors declare that the research was conducted in the absence of any commercial or financial relationships that could be construed as a potential conflict of interest.

## Publisher's note

All claims expressed in this article are solely those of the authors and do not necessarily represent those of their affiliated organizations, or those of the publisher, the editors and the reviewers. Any product that may be evaluated in this article, or claim that may be made by its manufacturer, is not guaranteed or endorsed by the publisher.
